# F114. DISORGANIZATION AND COGNITIVE DYSFUNCTIONS IN SCHIZOPHRENIA: A STUDY OF RESTING STATE EEG

**DOI:** 10.1093/schbul/sby017.645

**Published:** 2018-04-01

**Authors:** Ananrita Vignapiano, Thomas Koenig, Armida Mucci, Giulia Maria Giordano, Antonella Amodio, Giorgio Di Lorenzo, Cinzia Niolu, Mario Altamura, Antonello Bellomo, Silvana Galderisi

**Affiliations:** 1 University of Campania “L. Vanvitelli”; 2 Translational Research Center, University Hospital of Psychiatry Bern; 3 University of Rome ‘Tor Vergata’; 4 Psychiatry Unit, University of Foggia

## Abstract

**Background:**

A disorganization factor was found by several factor-analytic studies of schizophrenia symptoms. This factor does not appear to be affected by age, severity of other symptoms and chronicity of illness. A greater severity of disorganization is associated with poor functioning. Despite the general similarity of different factorial model, there is no consensus about which symptoms have to be included in the disorganization factor. Using the Positive and Negative Syndrome Scale (PANSS), Conceptual disorganization’ (P2), ‘Difficulty in abstract thinking’ (N5) and ‘Poor attention’ (G11) were core features of the disorganization factor.

The overlap of these items with neurocognitive functions is still debated. However, the heterogeneity of this factor and its neurobiological basis should be further investigated.

In the context of the multicenter study of the Italian Network for Research on Psychoses, the main aims of our study were to investigate electrophysiological and neurocognitive correlates of the disorganization factor, and to assess if each PANSS item, loading on the disorganization factor, could be underpinned by similar electrophysiological or cognitive alterations.

**Methods:**

Resting state EEGs were recorded for 5 minutes in 145 stabilized subjects with schizophrenia (SCZ) and 69 matched healthy controls (HC). The disorganization factor was evaluated using three PANSS items: P2, N5, and G11 (4).

Neurocognitive functions were assessed using the MATRICS Consensus Cognitive Battery (MCCB). Spectral amplitude was quantified in nine frequency bands. All statistical analyses of the scalp multichannel spectral amplitude (SAmp) data were performed using RAGU software.

Statistical comparisons between the SAmp maps of SCZ and HC were assessed by topographic analyses of variance (TANOVA). In SCZ, topographic analyses of covariance (TANCOVA) evaluated correlations between SAmp and disorganization, its constituent items and MCCB domains. Furthermore, Pearson’s correlations were performed between disorganization and its constituent items and MCCB neurocognitive domains.

**Results:**

TANOVA, comparing the group SAmp maps revealed increased Delta, Theta, and Beta1 and decreased Alpha2 SAmp in SCZ.

In the SCZ group, disorganization was significantly correlated to the Alpha1 SAmp. This relation was negative and most pronounced at occipital sites. At the items level, only N5 showed the same negative correlation at occipital sites.

MCCB neurocognitive composite score was associated with disorganization factor, and its constituent items P2 and N5. No significant correlation between Alpha1 SAmp and MCCB cognitive domains was observed.

**Discussion:**

Our findings illustrate the heterogeneity of disorganization dimension and a partial overlap with neurocognitive domains. ‘Difficulty in abstract thinking’ showed a unique association with Alpha1 activity, which is thought to be involved in the construction of conceptual maps.

Furthermore, the observed association of Alpha1 with ‘Difficulty in abstract thinking’ suggests that some aspects of disorganization could be underpinned by the impairment of basic neurobiological functions that are only partially evaluated using MCCB.

